# Expression of concern: Antibacterial and antibiofilm activities of silver-decorated zinc ferrite nanoparticles synthesized by a gamma irradiation-coupled sol–gel method against some pathogenic bacteria from medical operating room surfaces

**DOI:** 10.1039/d4ra90061h

**Published:** 2024-05-29

**Authors:** M. I. A. Abdel Maksoud, Gharieb S. El-Sayyad, Hanan S. El-Bastawisy, Rasha M. Fathy

**Affiliations:** a Materials Science Lab, Radiation Physics Department, National Center for Radiation Research and Technology (NCRRT), Egyptian Atomic Energy Authority (EAEA) Cairo Egypt; b Drug Microbiology Lab, Drug Radiation Research Department, National Center for Radiation Research and Technology (NCRRT), Egyptian Atomic Energy Authority (EAEA) Cairo Egypt adham_adham699@yahoo.com rashafathy82@gmail.com Gharieb.S.Elsayyad@eaea.org.eg

## Abstract

Expression of concern for ‘Antibacterial and antibiofilm activities of silver-decorated zinc ferrite nanoparticles synthesized by a gamma irradiation-coupled sol–gel method against some pathogenic bacteria from medical operating room surfaces’ by M. I. A. Abdel Maksoud *et al.*, *RSC Adv.*, 2021, **11**, 28361–28374, https://doi.org/10.1039/D1RA04785J.

*RSC Advances* is publishing this expression of concern in order to alert readers that concerns have been raised regarding the reliability of the SEM/EDX analysis in Fig. 13. An investigation is underway, and an expression of concern will continue to be associated with the article until a final outcome is reached.

Laura Fisher

22nd May 2024

Executive Editor, *RSC Advances*

## Supplementary Material

